# Tumor-Derived Exosomal miR-29b Reduces Angiogenesis in Pancreatic Cancer by Silencing ROBO1 and SRGAP2

**DOI:** 10.1155/2022/4769385

**Published:** 2022-10-14

**Authors:** Lihua Wang, Lei Yang, Tingting Zhuang, Xiuqing Shi

**Affiliations:** ^1^Department of Gastroenterology, Yantai Yuhuangding Hospital, #20 Yuhuangding East Road, Zhifu District, Yantai, Shandong 264000, China; ^2^Department of Internal Medicine, Yantai Yuhuangding Hospital, #20 Yuhuangding East Road, Zhifu District, Yantai, Shandong 264000, China; ^3^The General and Pediatric Surgery, Yantai Yuhuangding Hospital, #20 Yuhuangding East Road, Zhifu District, Yantai, Shandong 264000, China; ^4^Department of Medical Oncology, Yantai Yuhuangding Hospital, #20 Yuhuangding East Road, Zhifu District, Yantai, Shandong 264000, China

## Abstract

**Background:**

Exosomal miR-29b reportedly plays a role during cancer metastasis. However, its exact function and underlying mechanism during pancreatic cancer (PC) have not been investigated.

**Methods:**

Exosomes from PC cells were prepared and identified. Transmission electron microscopy (TEM) and confocal microscopy were used to examine structural characteristics of the exosomes and verify their internalization by human umbilical vein endothelial cells (HUVECs). The tube formation and migration abilities of HUVECs were detected. VEGF content was assessed by ELISA. GW4869 was used to suppress exosome release. Luciferase reporter assays were performed to verify the predicted interaction of miR-29b with ROBO1 and SRGAP2 mRNA.

**Results:**

Exosomal miRNA-29b was differentially expressed in the conditioned medium of PC cells. Exosomes from PC cells were verified by TEM and western blotting. Treatment with the exosomal inhibitor (GW4869) prevented an increase in miR-29b expression and recused the reduced VEGF expression and tube formation and migration abilities of HUVECs cocultured with BxPC3 and AsPC-1 cells that overexpressed miR-29b. Furthermore, the downregulation of ROBO1 and SRGAP2 in cocultured HUVECs was also reduced after additional treatment with GW4869. After incubation with miR-29b exosomes, HUVECs had lower VEGF concentrations and reduced migration and tube formation rates; however, those effects were eliminated by subsequent transfection with the miR-29b inhibitor. Luciferase reporter assays verified the interaction of miR-29b with ROBO1 and SRGAP2. That interaction was also supported by rescue assays showing that overexpression of ROBO1 and SRGAP2 also reduced the antiangiogenic effect of exosomal miR-29b in HUVECs.

**Conclusion:**

Exosomal miR-29b originating from PC cells protected HUVECs from PC cell-induced angiogenesis by attenuating ROBO1 and SRGAP2 expression. Our findings suggest a strategy for treating PC.

## 1. Introduction

Pancreatic cancer (PC) is one of the most frequently diagnosed and life-threatening neoplasms occurring in alimentary canals. Global cancer statistics for 2020 estimated there were 500,000 new PC cases and 460,000 deaths from PC, making PC the seventh leading cause of cancer death [1]. In 2025, PC is projected to overtake breast cancer as the third major reason for tumor-related death [2]. Due to its insidious symptoms, most PC patients are diagnosed with late-stage disease and have a poor prognosis [3]. The poor survival rate of PC patients with advanced stage disease can be attributed to tumor metastasis. Angiogenesis is responsible for advanced pancreatic carcinogenesis and enables tumor neovascularization to occur, which favors distant metastasis [4]. While preclinical studies of angiogenesis inhibitors have been conducted, the results have been unsatisfactory [5]. Therefore, fully deciphering the mechanism of PC angiogenesis remains an urgent priority.

MicroRNAs (miRNAs) are noncoding transcripts consisting of 18–23 nucleotides [6]. They are reported to regulate gene protein expression by binding to the 3′UTRs of mRNA molecules. In this way, miRNAs pleiotropically modulate gene functionality and are thereby implicated in various cellular functions, such as cellular growth and metabolic switching [7]. Exosomes are a family of extracellular vesicles with nanoscale sizes and are derived from various cells, including cancer cells [8]. They actively engage in molecular cross-talk and are involved in various physiopathological conditions, including sustained angiogenesis in cancer tissues [9]. For example, exosomes secreted by PC cells foster the recruitment of pancreatic stellate cells and stimulate distal metastases [10]. Exosomal miR-27a induces the angiogenesis of human microvascular endothelial cells [11]. Previous studies revealed that exosomal miR-29b attenuates oncogene behavior in lung cancer [12], colorectal cancer [13], and cervical cancer [14]. Exosomes derived from cancer-associated fibroblasts internalize miR-29b into hepatocellular carcinoma cells, where they negatively regulate cancer cell behavior [15]. miR-29b has been shown to be closely associated with angiogenesis-related factors [16], including pancreatic cancer. However, the effect of PC-derived exosomes and miR-29b on PC tumors remains unknown.

Convincing evidence has shown that miRNAs mediate PC cell malignant behavior by silencing the expression of target genes [17]. In this study, an online prediction by StarBase revealed that miR-29b sequences could bind to the 3′UTRs of ROBO1 (roundabout guidance receptor 1) and SRGAP2 (SLIT-ROBO Rho GTPase activating protein 2). The *ROBO1* gene is located on chromosome 3p12.3 and consists of 35 exons. It encodes an integral membrane protein that is a member of the immunoglobulin gene superfamily. *ROBO1* is reported to have an oncogenic function in certain malignancies. For example, the amplification of *ROBO1* causes chordoma cells to become invasive and metastasize [18]. In breast cancer, treatment with the anti-ROBO1 antibody reduces breast cancer-triggered angiogenesis and thereby retards cancer progression [19]. While Li et al. [19] reported the antitumorigenic function of ROBO1, ROBO1-driven tumor promotion has also been described [20, 21]. SRGAP2 is required to activate the GTPase activity of Rac. In hepatocellular carcinoma, an elevated level of SRGAP2 is an indicator of a poor prognosis, while SRGAP2 silencing drastically mitigates cancer metastasis. In contrast, SRGAP2 expression is downregulated in osteosarcoma and linked to an aggressive phenotype of that disease [22]. Therefore, the dual function of SRGAP2 in cancer is supported by experimental evidence. Researchers discovered that there is interaction between SRGAP2 and ROBO1. Considered that ROBO1 plays a key role in angiogenesis, and we hypothesized that SRGAP2 and ROBO1 coregulate angiogenesis in pancreatic cancer.

Here, we assumed that exosomal miR-29b from PC cells targeting ROBO1 and SRGAP2 might affect PC angiogenesis. Our findings reveal how miR-29b functions in PC angiogenesis and provide information useful for developing a novel drug for treatment of PC.

## 2. Methods

### 2.1. Cell Culture and Transfection

PC cells (BxPC3, PANC1, CFPAC-1, Capan-2, and AsPC-1), human umbilical vein endothelial cells (HUVECs), and a human normal pancreatic ductal epithelial cell line (HPDE6-C7) were purchased from the American Type Culture Collection (ATCC; Manassas, VA, USA). The BxPC3 and AsPC-1 cells were cultured in RPMI-1640 Medium (Thermo Fisher, Waltham, MA, USA), the PANC1 cells, HPDE6-C7 cells, and HUVECs were cultured in Dulbecco's Modified Eagle's Medium (Thermo Fisher, USA), the CFPAC-1 cells were cultured in Iscove's Modified Dulbecco's Medium (Thermo Fisher, USA), and the Capan-2 cells were cultured in McCoy's 5A Medium (Thermo Fisher, USA). All cells were cultured at 37°C in a 5% CO_2_ atmosphere.

miR-29b mimics, an miR-29b inhibitor, and mimic/inhibitor NC recombinant constructs that overexpressed ROBO1 or SRGAP2 (pCDNA-ROBO1 and pCDNA-SRGAP2) were purchased from Genepharm (Sunnyvale, CA, USA). Lipofectamine 2000 (Invitrogen, Waltham, MA, USA) was used to facilitate the introduction of the miRNA oligonucleotides and overexpressing vectors into BxPC3 and AsPC-1 cells. Cells were was pretreated with GW4869 at concentration 10 *μ*M (dissolved in DMSO) for 2 h prior to other treatments. RT-qPCR was performed to verify whether the transfections were successful.

### 2.2. Isolation and Characterization of Exosomes from BxPC3 and AsPC-1

An Exosome Isolation Kit (Denmark) was used to isolate exosomes (Exos/BxPC3 and Exos/AsPC-1) from BxPC3 and AsPC-1 cells per the manufacturer's instructions. Briefly, BxPC3 and AsPC-1 cells were cultured to 85% confluence in 6-well plates; after which, the cell supernatants were collected and exposed to Exosome Concentration Solution at 4°C. The mixture was then allowed to rest for 2 h at 4°C prior to centrifugation. The collected exosome pellets were purified using an Exosome Purification Filter and collected for subsequent use. For particle size measurement, the collected exosomes were resuspended in prechilled PBS and stained with 2% phosphotungstic acid (pH 6.8), and their morphology features were observed under a transmission electron microscope. The exosomes were also verified by western blotting with anti-TGS101 antibodies and anti-CD63 antibodies. The particle size of exosomes was also characterized by size distribution using particle size analyzer (N30E, NanoFCM).

### 2.3. Exosome Cellular Uptake

To verify the internalization of Exos/BxPC3 and Exos/AsPC-1 by HUVECs, we first labeled the exosomes by using a PKH26 Red Fluorescent Cell Linker Mini Kit (Merck, Rahway, NJ, USA) as instructed by the manufacturer. HUVECs (1 × 10 [5]) were seeded onto round coverslips of 18 mm diameter. Twenty-four hours later, the labeled exosomes were added for an additional 12 h of incubation. Next, the HUVECs were washed with PBS, fixed with 4% paraformaldehyde for 10 min, and then stained with DAPI for 30 min. Exosome uptake by the recipient HUVECs was visualized under a Nikon A1-R confocal microscope (Nikon Instruments, Tokyo, Japan).

### 2.4. RT-qPCR

Total cellular RNA was extracted using TRIzol Reagent (Invitrogen, USA) and then reverse transcribed into cDNA by using a iScript™ cDNA Synthesis Kit (Bio-Rad, Hercules, CA, USA) or miRNA 1st Strand cDNA Synthesis Kit (Vazyme, China). The resultant cDNA was quantified by SYBR Green Quantitative PCR (Roche, South San Francisco, USA) performed on a PCRmax Eco 48 thermal cycler (Thermo Fisher, USA). Fold-changes in target gene expression were analyzed by the Delta-Delta CT method. The following primers were used. miR-29b, forward primer: 5′-UAGCACCAUUUGAAAUCAGUGUU-3′, reverse primer: 5′-CACUGAUUUCAAAUGGUGCUAUU-3′; ROBO1, forward primer: 5′-CCCGACTTCACTCTCTCCCT-3′, reverse primer: 5′-AAATGGTGGGCTCAGGATGG-3′; SRGAP2, forward primer: 5′-TGAGATGGACTACTCCCGCA-3′, reverse primer 5′TGGTAGCCTAAGTCACAACACT3′; U6, forward primer: 5′-CTCGCTTCGGCAGCACA-3′, reverse primer: 5′-AACGCTTCACGAATTTGCGT-3′; and GAPDH, forward primer: 5′-TGTTCGTCATGGGTGTGAAC-3′, reverse primer: 5′-ATGGCATGGACTGTGGTCAT-3′.

### 2.5. Western Blotting

Cells were lysed with RIPA lysis buffer, and the total protein concentration in each supernatant was determined using a BCA Protein Assay Kit (Pierce Biotechnology, Waltham, MA, USA). Next, a 20 *μ*g sample of total protein from each supernatant was loaded onto a 12% SDS-PAGE gel and separated at 80 V for 40 minutes. The protein bands were then transferred onto PVDF membranes, which were subsequently blocked with 10% nonfat milk. Next, the membranes were incubated with anti-TSG101 antibodies (Cat#: BM4821, 1 : 1000, Boster, China), anti-CD63 antibodies (Cat#: PB9250,1 : 1000, Boster, China), anti-GRP94 antibodies (Cat#: PROTP14625, 1 : 1000, Boster, China), anti-ROBO1 antibodies (Cat#: A01530-2, 1 : 1000, Boster, China), anti-SRGAP2 antibodies (Cat#PA5-55792, 1 : 1000, Invitrogen, USA), and anti-GAPDH antibodies (Cat#A00227-1, 1 : 1000, Boster, China) at 4°C overnight. Next, the membranes were incubated with secondary antibodies at room temperature for an additional 1 h. The immunostained protein bands were visualized with Pierce ECL Western Blot Substrate (Merck, USA).

### 2.6. Enzyme-Linked Immunosorbent Assay (ELISA)

A Human VEGF ELISA Kit (Solarbio, China) was used to detect the VEGF concentrations in HUVECs according to the manufacturer's instructions. Briefly, the supernatant fractions of HUVECs were collected and spread across standard samples that had been precoated with goat anti-hamster IgG for 2.5 h at room temperature. Next, biotinylated VEGF detection antibodies were added for 1 h, HRP-Streptavidin solution was added for additional 45 min, and TMB One-Step Substrate Reagent was added for another 30 min. When the reaction was stopped, cell absorption was analyzed at 450 nm.

### 2.7. Tube Formation Assay

HUVECs were plated into 12-well plates (2 × 10^3^ cells per well) that had been precoated with BD Matrigel Basement Membrane Matrix (Bioscience, USA, San Francisco, CA, USA). BxPC3 and AsPC-1 cells were placed into the upper Transwell inserts, which allowed culture medium to flow into the Matrigel. Forty-eight hours later, a light microscope was used to view the capillary network.

To assess the impact of Exos/BxPC3 and Exos/AsPC-1 on HUVEC tube formation, the extracted exosomes were directly incubated with HUVECs. After 48 h of incubation, the amount of tube formation was recorded.

### 2.8. Transwell Migration Assay

Culture medium containing HUVECs was placed into the upper chambers of Transwell plates (2 × 10^3^ cells/chamber), and the lower Transwell chambers were filled with 500 *μ*L of culture medium containing 10% FBS and Exos/BxPC3 plus Exos/AsPC-1 or the conditioned medium. Twenty-four hours later, the upper inserts were removed and the migrated cells were fixed with 5% glutaraldehyde for 10 min. Next, 1% crystal violet in 2% ethanol was added to stain the migrated cells. Finally, the cells were visualized under a microscope.

### 2.9. Luciferase Reporter Assay

The StarBase website was used to predict the targets of miR-29b. The ROBO1 3′UTR and SRGAP2 3′UTR wild-type sequences predicted to interact with miR-29b, and also, and the corresponding mutant (MUT) sequences were amplified and fused into pGL3 luciferase reporter vectors to produce the following recombinant luciferase vectors: pGL3-ROBO1 3′UTR WT, pGL3-ROBO1 3′UTR MUT, pGL3-SRGAP2 3′UTR WT, and pGL3-SRGAP2 3′UTR MUT. The newly established vectors were introduced into HUVECs along with the miR-29b mimic or mimic NC. Quantification measurements of luciferase activity were obtained by using a luciferase reporter system (Promega, Madison, WI, USA).

### 2.10. Statistical Analysis

All statistical data were shown in mean ± SD and analyzed using the GraphPad Prism 8 software (GraphPad Software, Inc., La Jolla, CA, USA). Differences among multiple groups were analyzed by one-way ANOVA, followed by the Dunnett's post hoc test. A *P* value < 0.05 was considered to be statistically significant.

## 3. Results

### 3.1. Characterization of Exosomal miR-29b in PC Cells

**Figure 1:**
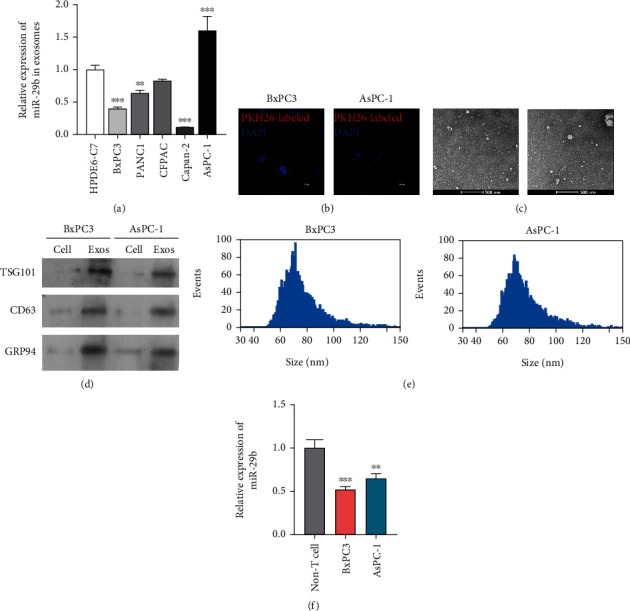
Identification of exosomal miR-29b originating from PC cells. (a) RT-qPCR analysis of miR-29b in exosomes extracted from HPDE6-C7, BxPC3, PANC1, Capan-2, and AsPC-1 cells. ^∗∗^*P* < 0.01 and ^∗∗∗^*P* < 0.001 vs. HPDE6-C7. (b) After coincubation with PKH26-labled exosomes, HUVECs were counterstained with a nuclear marker (DAPI) and viewed under a confocal microscope (200x). (c) TEM image of exosomes isolated from BxPC3 and AsPC-1 cells (8000x). (d) Western blot analysis of TSG101 and CD63 protein expression in exosomes derived from BxPC3 and AsPC-1 cells. Protein samples from BxPC3 and AsPC-1 cells served as negative controls. (e) Particle size of exosomes was analyzed. (f) RT-qPCR analysis of miR-29b expression in BxPC3 cells, AsPC-1 cells, and non-T cells. ^∗∗^*P* < 0.01 and ^∗∗∗^*P* < 0.001 vs. non-T cells. Experiments were repeated for three times and presented as mean ± SD.

After considering the antimetastatic and antiantigenic potentials of miR-29b in different cancers [23, 24], we sought to investigate the mechanism by which exosomes might participate in pancreatic carcinogenesis. To do this, we first assessed the universal expression of exosomal miR-29b in a panel of PC cells. We found that when compared to normal human pancreatic HPDE6-C7 cells, the PC cells (BxPC3, PANC1, Capan-2, and AsPC-1) all showed a differential expression of exosomal miR-29b (Figure 1(a)). It was noted that BxPC3 and AsPC-1 cells exhibited a relatively low or high metastatic ability [25] when miR-29b was expressed at a relatively low or high level, respectively. To avoid biased results, we used both types of cells for subsequent assays. After cocultivation with HUVECs, the exosomes extracted from BxPC3 and AsPC-1 cells were successfully transferred into the HUVEC cells, as evidenced by an aggregated red fluorescence surrounding the HUVEC nucleus (Figure 1(b)). As shown in Figure 1(c), a TEM imaging analysis was performed to visualize the typical cup-shaped appearance of exosomes from both types of PC cells. A western blot analysis revealed that CD63, TSG101, and GRP94 were highly expressed in PC cell-derived exosomes, but not in the cells (Figure 1(d)). As shown in Figure 1(e), the particle size of exosomes was ranging from 50 to 100 nm (Figure 1(e)). In contrast to the differential expression of miR-29b in PC-derived exosomes, miR-29b expression was significantly downregulated in BxPC3 and AsPC-1 cells (Figure 1(f)), suggesting a role for exosomal miR-29b during PC malignancy.

### 3.2. Exosomal miR-29b Transferred from PC Cells Reduced Angiogenesis *In Vitro*

**Figure 2:**
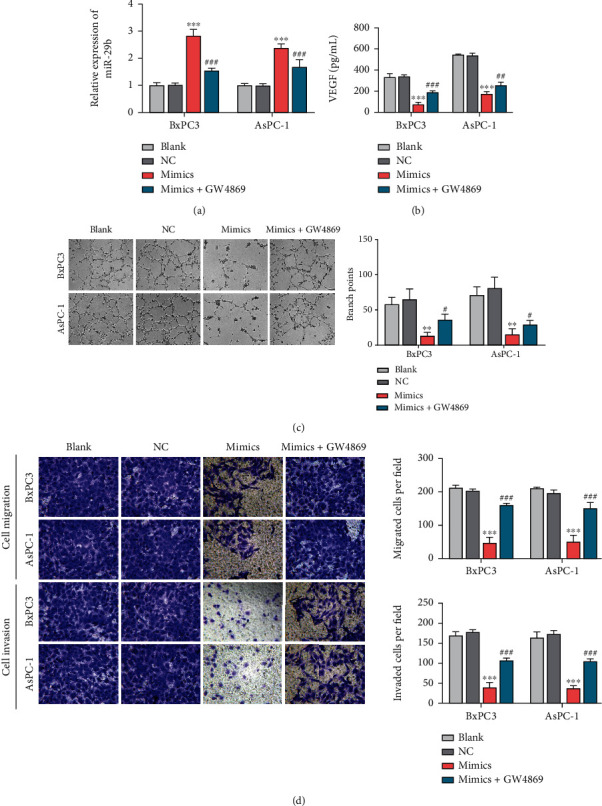
miR-29b-containing exosomes reduced HUVEC migration and tube formation. miR-29b mimics or mimic NCs were transfected into BxPC3 and AsPC-1 cells. Forty-eight hours later, both cell types were cocultivated with HUVECs for 24 h and then maintained in the presence or absence of GW4869 for an additional 48 h. (a) RT-qPCR analysis of miR-29b expression in HUVECs. HUVECs were stimulated in the coculture system with BxPC3 and AsPC-1 cells. After 48 h of stimulation, (b) VEGF content in the culture medium of HUVECs was assessed by ELISA. (c) Tube formation assays were conducted to determine HUVEC tube formation (100x). (d) Transwell migration assays were conducted to determine HUVEC migration (200x). ^∗∗^*P* < 0.01 and ^∗∗∗^*P* < 0.001 vs. NC; ^#^*P* < 0.05 and ^###^*P* < 0.001 vs. mimics. Experiments were repeated for three times and presented as mean ± SD.

Because miR-29b has been found to confer a defect in tumor cell-induced angiogenesis in several cancers [26, 27], we further investigated whether exosomes derived from PC cells might participate in miR-29b-mediated PC tumor suppression. To address that question, we transfected miR-29b mimics into BxPC3 and AsPC-1 cells and then cocultured the cells with HUVECs prior exposure to an exosome inhibitor (GW4869). The identification of exosomes has been shown in Figure S1. As shown in Figure 2(a), an accumulation of miR-29b in the HUVECs was verified; however, that significant increase in miR-29b expression was reduced by subsequent exposure to GW4869, indicating that miR-29b-containing exosomes had been received by the HUVECs. Furthermore, miR-29b enforced expression obviously reduced the levels of VEGF, a potent modifier of angiogenesis, while additional exposure to GW4869 partially rescued those reduced VEGF expression levels (Figure 2(c)). After cocultivation, HUVEC tube formation was inhibited by the miR-29b mimics, but that reduced tube formation ability was offset by subsequent treatment with GW4869 (Figure 2(d)). Likewise, miR-29b overexpression caused a reduction in HUVEC migration; however, that` reduction was eliminated by subsequent treatment with GW4869 (Figure 2(e)). Taken together, these findings indicate that miR-29b reduces angiogenesis by PC cells *in vitro* via exosome secretion.

### 3.3. PC Exosomal miR-29b Enhanced the Migration and Tube Formation Abilities of HUVECs

**Figure 3:**
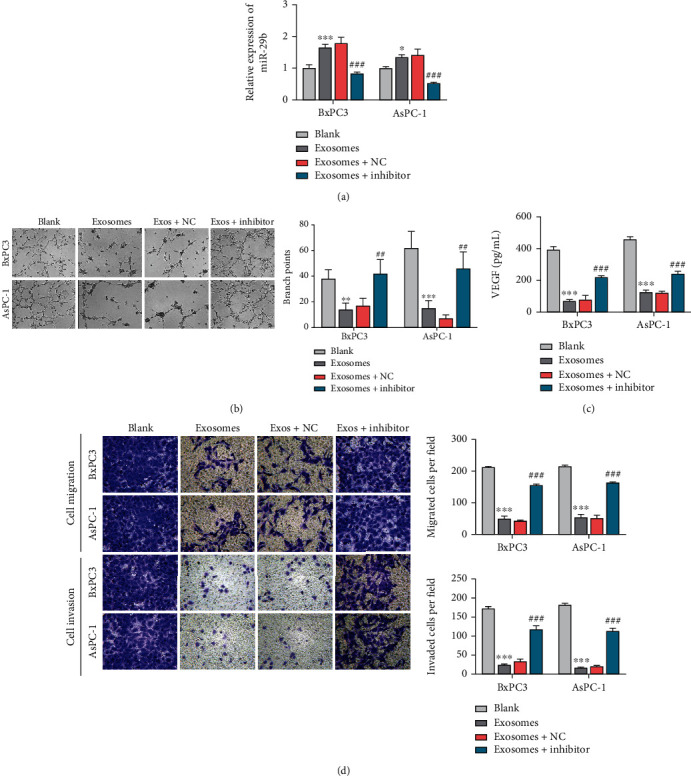
Exosomal miR-29b from PC cells enhanced HUVEC migration and tube formation. (a) RT-qPCR analysis of miR-29b expression in recipient HUVECs incubated with exosomes secreted from BxPC3 and AsPC-1 cells transfected with miR-29 mimics and also in recipient HUVECs treated with the miR-29b inhibitor or inhibitor NC. The parental HUVECs served as blank control cells. (b) Tumor formation assays were performed on HUVECs treated with exosomes, exosomes+miR-29 inhibitor, and exosomes+miR-29 inhibitor NC (100x). The parental HUVECs served as blank control cells. (c) ELISA detection of VEGF levels in HUVECs treated with exosomes, exosomes+miR-29 inhibitor, and exosomes+miR-29 inhibitor NC. The parental HUVECs served as blank control cells. (d) Transwell migration assays for determining the migration of HUVECs treated with exosomes, exosomes+miR-29 inhibitor, and exosomes+miR-29 inhibitor NC. The parental HUVECs served as blank control cells (200x). ^∗∗^*P* < 0.01 and ^∗∗∗^*P* < 0.001 vs. blank; ^##^*P* < 0.01 and ^###^*P* < 0.001 vs. exosomes+NC. Experiments were repeated for three times and presented as mean ± SD.

To further investigate the effects of exosomal miR-29 on tumor cell-induced angiogenesis, exosomes derived from BxPC3 and AsPC-1 cells transfected with miR-29 mimics (Exos/BxPC3^miR-29^ and Exos/AsPC-1 ^miR-29^) were incubated with HUVECs prior to treatment with the miR-29 inhibitor or inhibitor NC. As shown in Figure 3(a), a strong upregulation of miR-29 expression was detected in HUVECs transfected with Exos/BxPC3^miR-29^ or Exos/AsPC-1^miR-29^; however, that increase in miR-29 expression was eliminated after treatment with the miR-29 inhibitor, but not by treatment with the inhibitor NC. Furthermore, tube formation assays showed that HUVECs in the exosomal miR-29 group displayed reduced tube formation, which was rescued by subsequent treatment with the miR-29 inhibitor (Figure 3(b)). Moreover, ELISA results showed that the reduced VEGF levels in the culture medium of receipt HUVECs treated with Exos/BxPC3^miR-29^ or Exos/AsPC-1^miR-29^ could be rescued by treatment with the miR-29b inhibitor (Figure 3(c)), supporting the antiangiogenic effect of exosomal miR-29b on HUVECs. Similarly, the reduced HUVEC migration in the exosomal miR-29b group was also rescued along with miR-29 depletion (Figure 3(d)). In summary, exosomal miR-29 was found to be responsible for inhibition of angiogenesis during PC malignancy.

### 3.4. miR-29b Targeted ROBO1 and SRGAP2

**Figure 4:**
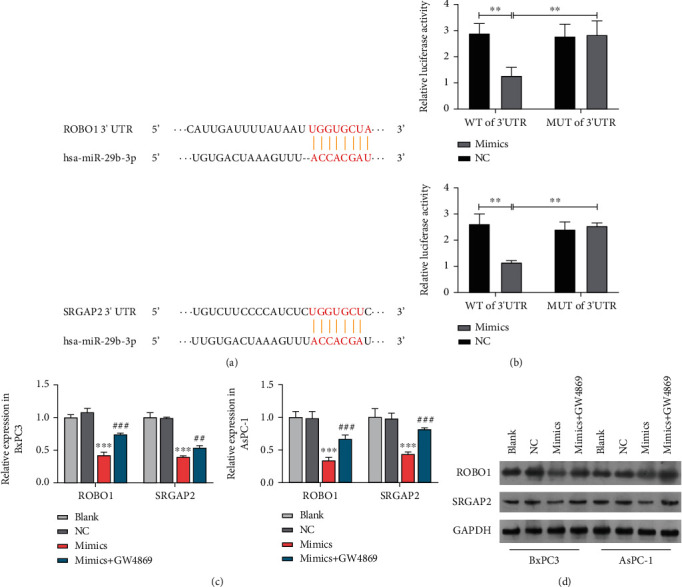
miR-29b targeted ROBO1 and SRGAP2. (a) Base pairings of the ROBO1 3′UTR and SRGAP2 3′UTR with miRNA-29b as verified by the StarBase website (http://starbase.sysu.edu.cn/). (b) Quantification of luciferase activity in HUVECs transfected with the ROBO1 3′UTR and SRGAP2 3′UTR to form pGL3-luciferase reporter constructs with the miRNA-29b mimic or mimic NC. (c) RT-qPCR analysis of ROBO1 and SRGAP2 expression in HUVECs incubated with miRNA-29b overexpressing BxPC3 and AsPC-1 cells that had been treated or not treated with GW4869. (d) Western blot study to determine the expression of ROBO1 and SRGAP2 proteins in HUVECs incubated with miRNA-29b overexpressing BxPC3 and AsPC-1 cells that had been treated or not treated with GW4869. ^∗∗∗^*P* < 0.001 vs. NC; ^###^*P* < 0.001 vs. mimics+GW489. Experiments were repeated for three times and presented as mean ± SD.

To decipher the mechanism behind the antiangiogenic effect of exosomal miR-29b in HUVECs, we used StarBase to search for possible miR-29b targets based on complimentary mRNA 3′UTR sequences. As shown in Figure 4(a), the ROBO1 3′UTR and SRGAP2 3′UTR matched 8 nucleotides of miR-29b. After fusing the wild-type (WT) and mutated (MUT) sequences of the ROBO1 3′UTR and SRGAP2 3′UTR into pGL3-luciferase reporter constructs, we cotransfected the resultant WT or MUT constructs into HUVECs treated with the miR-29b mimic or mimic NC. Considerably less luciferase activity resulting from the ROBO1 3′UTR WT and SRGAP2 3′UTR WT was observed in HUVECs transfected with the miR-29b mimics, while no significant change was detected in HUVECs cotransfected with the mimic NC (Figure 4(b)), suggesting that miR-29b targeted the ROBO1 3′UTR and SRGAP2 3′UTR. To verify this finding, we detected the expression of ROBO1 and SRGAP2 in recipient HUVECs that were coincubated with BxPC3 and AsPC-1 cells with or without GW4869 treatment. As shown in Figures 4(c) and 4(d), GW4869 treatment rescued the reduced expression of ROBO1 and SRGAP2 in recipient HUVECs that had been cocultured with BxPC3 and AsPC-1 cells transfected with miR-29 mimics, suggesting that exosomes containing miR-29b mimics simultaneously reduced ROBO1 and SRGAP2 expression in the HUVECs.

### 3.5. miR-29b Reduced HUVEC Migration and Tube Formation by Downregulating ROBO1 and SRGAP2

**Figure 5:**
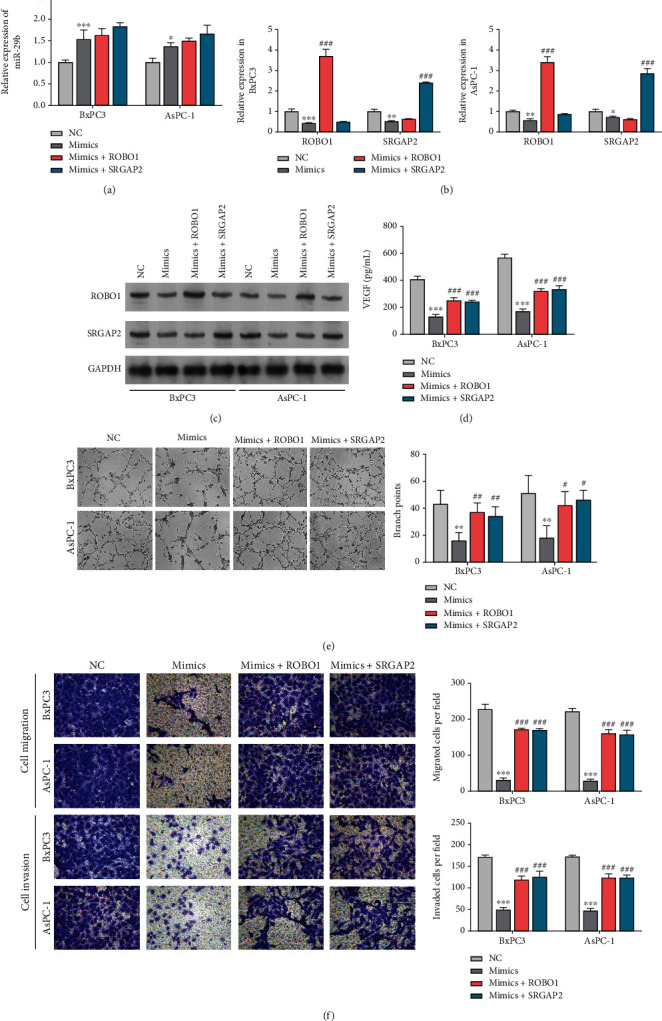
Exosomal miR-29b reduced HUVEC migration and tube formation by downregulating ROBO1 and SRGAP2. (a) RT-qPCR analysis of miR-29b expression in recipient HUVECs incubated with exosomes secreted from BxPC3 and AsPC-1 cells transfected with ROBO1 or SRGAP2 overexpressing constructs. (b) RT-qPCR analysis of ROBO1 and SRGAP expression in recipient HUVECs that had been incubated with exosomes secreted from BxPC3 cells transfected with ROBO1 or SRGAP2 overexpression constructs. (c) Western blot assessment of SRGAP2 and ROBO1 protein expression in recipient HUVECs incubated with exosomes secreted from AsPC-1 cells transfected with ROBO1 or SRGAP2 overexpressing constructs. (d) ELISA detection of VEGF levels in HUVECs incubated with exosomes secreted from the AsPC-1 cells transfected with ROBO1 or SRGAP2 overexpressing constructs. (e) Tube formation assay to assess the tube formation ability of HUVECs incubated with exosomes secreted from AsPC-1 cells transfected with ROBO1 or SRGAP2 overexpressing constructs (100x). (f) Transwell migration assay to assess the migration ability of HUVECs incubated with exosomes secreted from AsPC-1 cells transfected with ROBO1 or SRGAP2 overexpressing constructs (200x). ^∗∗^*P* < 0.01 and ^∗∗∗^*P* < 0.001 vs. NC; ^#^*P* < 0.05, ^##^*P* < 0.01, and ^###^*P* < 0.001, vs. mimics. Experiments were repeated for three times and presented as mean ± SD.

Having demonstrated that exosomal miR-29b from PC cells downregulated ROBO1 and SRGAP2 expression in HUVECs, we investigated whether ROBO1 and SRGAP2 were necessary for suppression of tumor cell-induced angiogenesis by exosomal miR-29b. Recombinant constructs that overexpressed ROBO1or SRGAP2 were delivered into HUVECs incubated with Exo/BxPC3^miR-29b^ and Exo/AsPC-1^miR-29b^. A quantitative increase in miR-29b expression in the recipient HUVECs indicated that miR-29b had been internalized (Figure 5(a)). As anticipated, the internalization of miR-29b greatly reduced the expression of ROBO1 and SRGAP2, but both reductions in expression were recovered after transfection with the recombinant constructs overexpressing ROBO1 or SRGAP2 (Figure 5(b)). Western blot studies conducted to detect ROBO1 or SRGAP2 protein expression (Figure 5(c)) further confirmed that miR-29b was not needed for downregulation of ROBO1 and SRGAP2 in HUVECs. Functionally, the reduced expression of VEGF in HUVECs incubated with Exo/BxPC3^miR-29b^ and Exo/AsPC-1^miR-29b^ was rescued along with ROBO1 and SRGAP2 overexpression (Figure 5(d)). Moreover, the decreased migration and tube formation abilities of HUVECs with Exo/BxPC3^miR-29b^ and Exo/AsPC-1^miR-29b^ were mitigated by overexpression of ROBO1 or SRGAP2 (Figures 5(e) and 5(f)). Taken together, these results indicated that exosomal miR-29b from BxPC3 and AsPC-1 cells reduced angiogenesis *in vitro* by downregulating ROBO1 or SRGAP2.

## 4. Discussion

Although angiogenesis is involved in PC malignancy [28], drugs that target angiogenesis have produced limited benefits in patients with PC [4]. Therefore, an in-depth exploration of the underlying mechanism of PC angiogenesis is required. In this study, we found that exosomal miR-29b considerably reduced HUVEC migration and angiogenesis by targeting ROBO1 and SRGAP2.

There is compelling evidence for the importance of cell-to-cell cross-talk facilitated by tumor-derived exosomes during tumor-induced angiogenesis [29]. For example, exosomes derived from PC cells exposed to hypoxic conditions promote angiogenesis by transferring lncRNA UCA1 into HUVECs [3]. miR-29b has been reported to be downregulated in PC and serves as an antitumorigenic miRNA by inhibiting tumor growth and metastatic dissemination [30–32]. Zeng et al. [33] reported the association between a high level of miR-29b expression and a better prognosis for PC patients [33]. Consistent with previous investigations, we found that miR-29b expression was decreased in PC cells, while exosomal miR-29b displayed differential expression in the conditioned medium of PC cells. Furthermore, the exosomes had been internalized by HUVECs, supporting subsequent efforts to understand their role in PC-induced angiogenesis. We found that the unregulated levels of miR-29b expression in HUVECs coincubated with BxPC3 and AsPC-1 cells overexpressing miR-29b could be reduced by GW4869. In addition to the decreased accumulation of miR-29b in HUVECs treated with GW4869, our studies of HUVEC tube formation and migration abilities, coupled with the effects of a potent angiogenesis stimulator (VEGF), showed that the inhibition of HUVEC angiogenesis by exosomes from PC cells transfected with miR-29b mimics could be reversed by GW4869. This suggested that an exosome complex containing miR-29b contributed to tumor suppression. Consistent with the above results, we also found that exosomal miR-29b inhibited PC-induced angiogenesis in HUVECs, and those reductions could be rescued by the miR-29b inhibitor. Our findings further support an antitumorigenic role for miR-29b during PC malignancy.

Canonically, miRNAs exert the effect by binding to sequences in the 3′UTRs of mRNA molecules [34]. Our data showed that miR-29b targeted ROBO1 mRNA and SRGAP2 mRNA. ROBO1 and SRGAP2 expression were both reduced in HUVECs incubated with BxPC3 and AsPC-1 cells that overexpressed miR-29b; however, those reductions were rescued by exposure to GW4869, suggesting that an exosome complex carrying miR-29b repressed ROBO1 and SRGAP2 expression. Consistent with those findings, the impaired expression of ROBO1 and SRGAP2 caused by exosomal miR-29b was also recovered by the miR-29b inhibitor, highlighting the interaction of miR-29b with ROBO1 and SRGAP2. Previous studies showed that ROBO1 increases PC cell proliferation, migration, and invasion and thereby promotes tumor growth [18–21]. Furthermore, a microarray study revealed that a high level of ROBO1 expression was associated with PC lymphatic metastasis [35].

While an unregulated level of ROBO1 expression in PC tumor stroma was found to support tumor invasiveness and metastasis [36], ROBO1 overexpression in PC cells (PANC-1 and MiaPaca-2) was found to reduce cell proliferation, suggesting a tumor suppressive effect [37]. Our data for HUVECs containing exosomal miR-29b showed that ROBO1 overexpression could rescue a decrease in VEGF expression, as well as decreases in cell migration and tube formation after miR-29b mimic transfection. Therefore, our data further support the oncogenic role of ROBO1 during PC progression. The discrepancy regarding the role played by ROBO1 in PC might be associated with cell-context. Furthermore, the role played by SRGAP2 in PC has not been described. Our data showed that ROBO1 overexpression reversed the antiangiogenic effect on HUVECs caused by exosomal miR-29b. While SRGAP2 is described as an oncogenic gene in hepatocellular carcinoma [38], it functions a metastasis suppressor in osteosarcoma [22]. Therefore, our findings provide further evidence of a context-dependent role for SRGAP2 during cancer progression.

In conclusion, our study revealed for the first time that exosomal miR-29b secreted by PC cells inhibits angiogenesis by HUVECs by targeting SRGAP2 and ROBO1. Our data provide a theoretical basis for the use of exosomes in PC intervention. However, *in vivo* studies are also required to further address the *in vivo* role of exosomal miR-29b during PC progression. At the same time, there are limitations in this study. For example, angiogenesis-related factors were not examined in clinical samples, and we will explore in depth in subsequent studies. Besides, an animal experiment should be included in further exploration.

## Data Availability

The data used to support the findings of this study are included within the article.

## Supplementary Materials

Supplementary Materials Figure S1: BxPC3 and AsPC-1 cell-derived exosomes were identified by using particle size analyzer and TEM.
