# Supplementary material for: Tumor-Derived Exosomal miR-29b Reduces Angiogenesis in Pancreatic Cancer by Silencing ROBO1 and SRGAP2
Source: J Immunol Res. 2022 Oct 14;2022:4769385. doi: 10.1155/2022/4769385 (PMC9586796; doi:10.1155/2022/4769385)
Supplement: Supplementary Materials — Figure S1: BxPC3 and AsPC-1 cell-derived exosomes were identified by using particle size analyzer and TEM. [file 4769385.f1.docx]

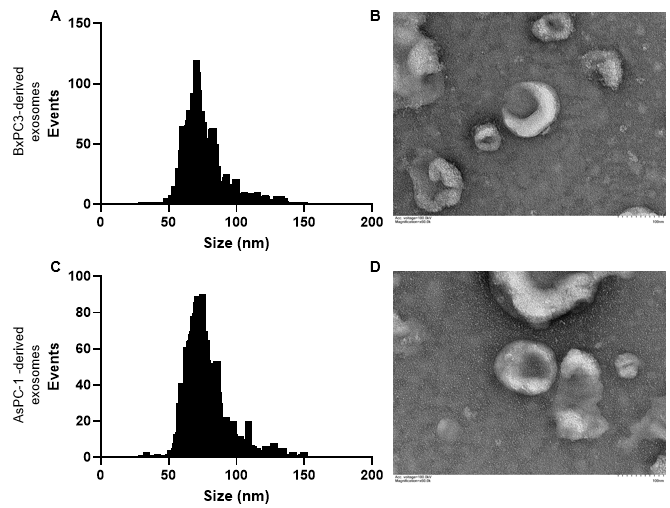


Figure S1. BxPC3 and AsPC-1 cells derived exosomes were identified by using particle size analyzer and TEM.
